# Lower extremity kinematic coupling during single and double leg landing and gait in female junior athletes with dynamic knee valgus

**DOI:** 10.1186/s13102-021-00385-y

**Published:** 2021-12-09

**Authors:** Mahdis Dadfar, Rahman Sheikhhoseini, Mohadeseh Jafarian, Alireza Esmaeili

**Affiliations:** 1 Department of Corrective Exercise and Sport Injury, Faculty of Physical Education and Sport Sciences, Allameh Tabataba’i University, Western Azadi Sport Complex Boulevard, Hakim Highway, Tehran, Iran; 2 Department of Electrical Engineering, Faculty of Electrical Engineering, Sharif University of Technology, Tehran, Iran; 3 Institute for Health and Sport (iHeS), Victoria University, Melbourne, Australia; 4 Australian Football League (AFL), Melbourne, Australia

**Keywords:** Knee joint, Kinetic chain, Cross-correlation, Vector coding, Biomechanics

## Abstract

**Background:**

Dynamic knee valgus (DKV) is a common lower extremity movement disorder among females. This study aimed to investigate kinematic couplings between lower extremity joints in female junior athletes with DKV during single and double-leg landing and gait.

**Methods:**

Twenty-six physically active female junior athletes (10–14 years old) with DKV were recruited. Kinematic couplings between rearfoot, tibia, knee, and hip were extracted using eight Vicon motion capture cameras and two force plates. Zero-lag cross-correlation coefficient and vector coding were used to calculate kinematic couplings between joints during physical tasks. Paired t-test and Wilcoxon tests were run to find significant couplings between joint motions and coupling strengths. Bonferroni posthoc was used to determine significance with α ≤ 0.05.

**Results:**

The results showed that the strongest kinematic relationship existed between rearfoot eversion/inversion and tibial internal/external rotation during all three tasks. Correlations of the rearfoot supination/pronation with tibial rotations, knee, and hip motions in sagittal, frontal, and transverse planes were very strong to strong during double-leg landing and moderate to weak during gait. A weak correlation was observed between rearfoot supination/pronation and hip adduction/abduction during single-leg landing.

**Conclusions:**

Coupling relationships between rearfoot, knee, and hip vary by the task intensity and alignment profiles in female juniors with DKV.

## Background

Dynamic knee valgus (DKV) has been reported as one of the contributing factors to Anterior Cruciate Ligament (ACL) injuries [1, 2]. This condition is described as a combination of changes in the knee joint, hip joint, and foot complex [3, 4]. Based on the previous findings, female athletes are more inclined to land with DKV than men, putting them at greater risk of ACL injuries [5, 6]. This tendency is partially explained by the typically lower levels of neuromuscular control in the hip joint in females compared to males [4, 7]. As another difference in their biomechanical characteristics, female athletes have demonstrated greater ankle eversion during jump-landing tasks, contributing to increased injury risk [8]. Moreover, previous findings indicated that ACL injuries due to gender-related factors start to occur around the age 12 {Shea, 2004 #3763}. The main potential reasons behind a greater rate of ACL strain in female athletes may be the neuromuscular imbalances [9], and the altered biomechanical characteristics such as excessive knee valgus [4, 10, 11], musculoskeletal and hormonal changes during pubertal development [12]. In addition to these changes, a previous study indicated altered motions of ankle joint in the frontal plane during an unanticipated cutting task in young female athletes [8].

Generally, foot and relatively ankle positions play an important role in the movements of the proximal joints in the frontal and transverse planes [13, 14]. For instance, rearfoot eversion and subtalar pronation may increase internal tibial rotation through the talocrural joint during the stance phase of running [15–17]. The reason behind the mentioned link between rearfoot and proximal segments’ motions, especially tibial rotations, can be related to the oblique orientation of the subtalar joint connected to the talocrural joint [18, 19]. For instance, it is observed that rearfoot inversion/eversion may be related to the altered hip motions at frontal and transverse planes, such as hip adduction [20, 21].

Regarding this biomechanical relationship between foot motions affecting the hip and knee joints, the kinematic analysis of unilateral standing indicated that excessive calcaneus eversion impelled by wedges under the foot may induce increased hip internal rotation and flexion and greater axial rotatory motion in the weight-bearing leg [22]. However, coupling motions between lower extremity joints do not always follow the same pattern and vary as the complexity and intensity of tasks change [23]. This coupling term is referred to as the rotational axis motions related to the motions in another joint [19]. Analyzing kinematic coupling of the lower extremity joints can provide additional information by studying joint kinematic behavior relative to its proximal/distal joints, compared to the routine joint kinematics evaluation [19]. This further information can be helpful in improving our understanding of the complex and interdependent changes in the exhibited joint reaction moment and forces in response to kinematic coupling strategies that may affect risk of sustaining ACL injuries [8].

Comparing kinematic coupling between joints in different activities from walking to jump-landing showed that in more physically demanding tasks, individuals exhibit greater femoral internal rotation due to the increased tibial internal rotation, which is found as a compensatory movement strategy [19]. Therefore, greater correlations between rearfoot and knee and hip joints may be observed in single-leg landing compared to the gait [20]. Moreover, lack of movement flexibility and coupling variability has been suggested as possible reasons behind some of the injuries in the musculoskeletal system [24]. In other words, the presence of coupling variability between joints reflects a flexible movement strategy in reacting to external perturbations and the subsequent fine motion adjustments over short time intervals, which may prevent injuries [23, 25, 26]. However, it is noteworthy to mention that if the knee joint shows greater kinematic motion to compensate limited hip joint engagement, knee joint is positioned at a less protected condition with greater joint loadings. This coupling knee-bias strategy is reported to increase the risks of knee joint injuries during physically intense activities such as cutting maneuvers and stop-jumping task frequently repeated in some sports such as basketball [27, 28]. While the kinematic coupling has shown to be task-dependent, a previous study reported limited ankle dorsiflexion motion to cause knee joint to go in greater hyperextension and cause greater stress on the knee joint [29].

One of the reasons behind the greater stress observed in the knee joint following changes in the distal joints motions can be the altered forces and moments in the joint as well as the muscular activation patterns [8]. More specifically, while a combination of increased tibial internal rotation and excessive knee valgus imparts the highest rates of shearing forces to the ACL, the influence of altered biomechanics between the ankle and knee joints also requires due attention [30, 31]. On the other hand, a combination of tibial external rotation and knee valgus may result in the ACL impinging on the femur epicondyle, putting extra stress on this ligament [13]. Given the biomechanical differences between male and female athletes [32], imbalanced alignments like DKV can contribute to injuries by altering the coupling kinematics during different physical tasks. Female athletes with DKV exhibit greater hip adduction and smaller rearfoot eversion during running, which may be considered a compensatory movement pattern in such activity [16].

Additionally, biomechanical characteristics such as impact forces and vertical stiffness differ between adolescents and adults [33]. Female athletes are also more inclined to develop altered biomechanical characteristics such as knee abduction angle and moment during puberty, increasing the risk of ACL injuries [7, 34]. Such age-related biomechanical differences are deemed to be the consequences of insufficient neuromuscular adaptations to the rapid growth of the skeletal system during puberty [35]. Although previous studies indicated that there is less coupling variability between knee flexion/extension and knee and hip rotation kinematics in female athletes compared to their male counterparts, evidence on coupling kinematics during different landing and gait tasks in female athletes with DKV or the ones during their pubertal growth is scarce [8, 19].

Despite the importance of coupling motions between rearfoot, knee, and hip joints in the context of injuries and the known biomechanical differences between genders, no studies to date have evaluated such motion couplings in young female athletes with DKV. Therefore, this study aimed to evaluate the cross-correlations between rearfoot, knee, and hip joints and coupling strengths during single-leg landing, double-leg landing, and gait in young female athletes with DKV.

## Methods

### Participants

Seventy female athletes were invited to this study. A single-leg squat test was used to determine the existence of DKV in the volunteers [36]. This test is a reliable tool for scoring knee joint motions in the frontal plane with intrarater and interrater reliability from 0.88 to 0.98 and 0.97 to 1.00, respectively [37]. In the next step, twenty-six participants who scored at least two out of three repetitions with noticeable valgus (defined as patella pointing towards the second toe) in both legs during single-leg squat were selected for the study. Sample size were estimated based on the Coefficient of Determination p^2^, the effect size of 0.78, α = 0.5 and power (1 − β) = 0.95 according to previous studies [23, 38, 39] by using G*Power software version 3.1. However, the sample size of the current study was strengthened to improve the precision and power of the estimations. The inclusion criteria were: female athletes with DKV, age between 10–14 years, 3–5 years of volleyball or basketball regular training experience. The exclusion criteria were: the presence of any pain in the spine or lower extremity, history of musculoskeletal injuries in the last year or fracture in lower extremities, cardiovascular diseases, balance impairments, history of surgery on spine or lower extremities, current use of any medications, and presence of any musculoskeletal malalignments based on the New York test. Demographic data for the participants of this study were: n = 26, age = 12.2 ± 2.0 years, body mass = 42.9 ± 11.4 kg, height = 154 ± 11 cm, BMI = 18.9 ± 2.2. The current study conformed to the Declaration of Helsinki, and was approved in terms of ethical considerations by the Committee for Ethics in Biomedical Research of the University of Social Welfare and Rehabilitation Sciences after obtaining the ethics code (IR.USWR.REC.1398.007), and all methods were performed in accordance with relevant guidelines and regulations. Since participants of this study were under the legal age, the informed consent was obtained from a parent and/or legal guardian of all the participants.

### Procedures

Eight Vicon motion capture cameras (120 Hz—2.2 megapixel Vero model cameras—UK) were used to record kinematic data, and two synched embedded in-floor Kistler force plates (1200 Hz—model 9286ba – 40 cm × 60 cm dimension – Switzerland) were used to record kinetic data during landing tasks and gait. Motion capture cameras and force plates were calibrated based on the Vicon system manufacturer recommendations. The raw data collection and processing were performed using the recommended plug-in-gait method by Vicon and Nexus software (version 2.9). The examiner determined each participants’ dominant leg by performing a shooting-questionnaire test before the laboratory tests [40]. A single examiner with sufficient training and experience conducted all the procedures.

#### Single-leg and double-leg landing tests

Participants stood on the dominant leg on a 30 cm box located 70 cm behind the center of the force plate. The non-dominant leg was flexed 90° at the knee joint. Participants were asked to keep their hands on their waist during the whole test procedure and then jump from the box on cue and land on the dominant leg while keeping the knee of the non-dominant leg flexed. Participants were asked to maintain their balance after landing for five seconds. A similar procedure was used for the double-leg landing test, with the only difference being the participants landing on both feet. In a successful landing movement individuals were required to land downwards without an upward or forward jumping pattern in the center of the force plate and not on the corners steadily without any wobbles, and were asked to maintain their landing posture for 5 s. Moreover, the correct landing test was done with forward-looking posture and without looking down on the force plate, and with arms placed on the waist without arm swings during the whole landing procedure. Both landing tests were repeated three times [20, 41].

#### Gait test

Participants were asked to walk comfortably on the walkway at their preferred speed and repeated the task three times. A successful gait trial participants were asked to perform the test at their preferred speed, without unnaturally managing their steps on the force plates with a natural arm swing and without looking downwards to the ground or force plates. Moreover, a gait trial was correct when performed with full heel strike on the center of the force plates and not the corners, and with right foot placement on the first and the left foot on the second force plates.

### Data collection

**Fig. 1:**
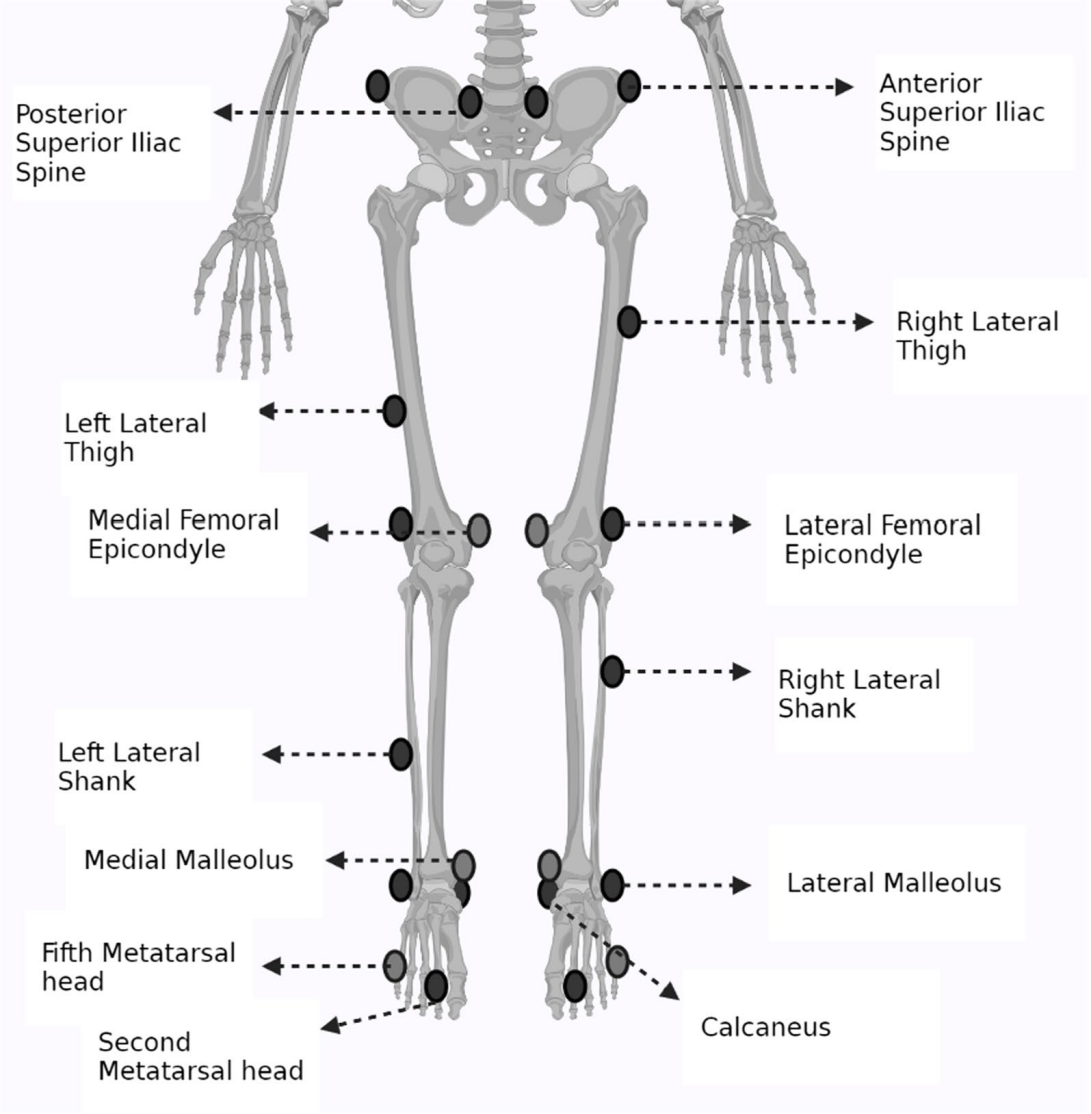
Lower extremity marker placement: 20 retroreflective markers based on the modified plug-in-gait marker system. These markers were placed laterally on posterior superior iliac spine, anterior superior iliac spine, lateral thigh, lateral femoral epicondyle, lateral shank, lateral malleolus, second metatarsal head, and calcaneus, the fifth metatarsal, medial malleolus, and medial femoral epicondyle

Twenty-two retroreflective markers were placed on lower extremity landmarks based on the Plug-in-Gait marker system: laterally on posterior superior iliac spine, anterior superior iliac spine, lateral thigh, lateral femoral epicondyle, lateral shank, lateral malleolus, second metatarsal head, and calcaneus. For anthropometric assessment, additional markers were placed on the fifth metatarsal, medial malleolus, and medial femoral epicondyle [42] (Fig. 1). Woltring filter (MSE10) was used to smooth and filter data under 20 frames [43]. The Euler angles in the joint frame during gait and landing tasks were determined for flexion/extension (x-axis), adduction/abduction (y-axis), and internal/external rotations (z-axis), based on the Vicon plug-in-gait model [44]. The extracted anthropometric data that were assessed in the static position were then applied to the dynamic data. The data of this study was extracted from the participants’ dominant leg generated from initial contact (IC) to maximum knee flexion (MAX) during both single-leg and double-leg landing tests and between heel-strike to toe-off of the same foot during the stance phase of the gait cycle, which were determined from the vertical ground reaction force (VGRF) curve. The biomechanical procedures and mean values were calculated using a self-developed code in MATLAB (version R2017b). Initial contact moment was defined as the time point when the VGRF threshold exceeded 10 N during each task [45]. The current study recorded and calculated the kinematic couplings between lower extremity joints in frontal and transverse motion planes. The cross-correlation method was used to calculate the couplings between rearfoot and knee and hip joints [21], which is a recommended approach for evaluating the kinematic relationships between different joints [46, 47]. In this method, the strengths of cross-correlation coefficients were calculated from the angular displacement curves of the two involved joints. Using this approach, the researcher could analyze the strength of the temporal link between joints’ angular data over two time points. Moreover, cross-correlation method provides the advantage of comparing two relative motions of two joints in the similar or different motion planes and thus, the kinematic coupling variability between two joints. The kinematic relationship between two joints was also defined using zero-lag normalized cross-correlation based on previously developed formulas [20, 21]. The coupling coefficient was calculated for the following angular data: rearfoot eversion (EVE)/inversion (INV) and hip flexion (FLX)/ extension (EXT), rearfoot EVE/INV and hip adduction (ADD)/abduction (ABD), rearfoot EVE/INV and hip internal rotation (INR)/external rotation (EXR), rearfoot pronation (PRO)/supination (SUP) and hip FLX/EXT, rearfoot PRO/SUP and hip ADD/ABD, rearfoot PRO/SUP and hip INR/EXR, rearfoot EVE/INV and knee FLX/EXT, rearfoot EVE/INV and knee adduction (ADD)/abduction (ABD), rearfoot EVE/INV and internal tibial rotation (INR)/external rotation (EXR), rearfoot PRO/SUP and knee FLX/EXT, rearfoot PRO/SUP and knee ADD/ABD, rearfoot PRO/SUP and tibial INR/EXR, ankle dorsiflexion (DOR)/ plantarflexion (PLAN) and hip FLX/EXT, ankle DOR/PLAN and knee FLX/EXT. The coupling strength between two joints in different tasks was defined based on the calculated correlation scores presented in previous studies [20, 48]. Vector coding (coupling angle) was used to interpret the quantity of the magnitude of the angular excursion and kinematic coupling in the involved joints and to determine the angular displacements between the proximal and distal joints [49]. The coupling angles between the proximal and distal joints were calculated from IC to MAX landing, and from heel contact to toe off during gait. The correlation strength was defined based on the correlation coefficients as follows: 1-Very strong (0.80 to 1.00 or -0.80 to -1.00), 2- Strong (0.60 to 0.79 or -0.60 to -0.79), 3- Moderate (0.40 to 0.59 or -0.40 to -0.59), 4- Weak (0.20 to 0.39 or -0.20 to -0.39), 5- Very weak (0 to 0.19 or 0 to -0.19) [50].

### Statistical analysis

Statistical analysis was conducted using IBM SPSS Version 24. Shapiro–Wilk's test was run to examine the data normality distribution. P-values less than 0.05 were considered statistically significant. During different tasks, the paired t-test and Wilcoxon tests were run to evaluate the significant differences in correlations, coupling strength, and coupling angle. Bonferroni posthoc test was performed to the significance level (α ≤ 0.05). The correlation strength was defined based on the correlation coefficients as follows: 1—Very strong (0.80 to 1.00 or − 0.80 to − 1.00), 2—Strong (0.60 to 0.79 or − 0.60 to − 0.79), 3—Moderate (0.40 to 0.59 or − 0.40 to − 0.59), 4—Weak (0.20 to 0.39 or − 0.20 to − 0.39), 5—Very weak (0 to 0.19 or 0 to − 0.19) [50].

## Results

### Single leg landing

Rearfoot PRO/SUP had a weak correlation with hip ADD/ABD, and rearfoot EVE/INV moderately correlated with hip ADD/ABD (*P* < 0.001 for all variables). Rearfoot motions at both frontal and transverse planes had very strong correlations with tibia INR/EXR, knee ADD/ABD, and hip INR/EXR. Moreover, ankle DOR/PLAN showed a strong correlation with hip and knee FLX/EXT, while very strong correlations were observed between rearfoot EVE/INV and PRO/SUP and hip and knee FLX/EXT (Table 1).

**Table 1:** Correlation mean coefficient scores + (SD) during different tasks for rearfoot, ankle, tibia, knee and hip motions in sagittal, frontal and transverse motion planes

Couplings	SLL N = 26	DLL N = 26	Gait N = 26
Rearfoot EVE/INV and hip FLX/EXT	0.86* (0.03)	0.87* (0.05)	− 0.2**** (0.08)
Rearfoot EVE/INV and hip ADD/ABD	0.48*** (0.37)	− 0.68** (0.56)	0.43*** (0.57)
Rearfoot EVE/INV and hip INR/EXR	0.80* (0.49)	0.95* (0.59)	0.29**** (0.73)
Rearfoot PRO/SUP and hip FLX/EXT	− 0.88* (0.02)	− 0.93* (0.02)	0.25**** (0.07)
Rearfoot PRO/SUP and hip ADD/ABD	− 0.23**** (0.40)	0.86* (0.48)	− 0.46*** (0.56)
Rearfoot PRO/SUP and hip INR/EXR	− 0.82* (0.51)	− 0.90* (0.65)	− .0.32**** (0.72)
Ankle DOR/PLAN and hip FLX/EXT	0.73** (0.02)	0.78** (0.02)	− 0.51*** (0.06)
Rearfoot EVE/INV and knee FLX/EXT	0.90* (0.03)	0.89* (0.05)	0.1***** (0.1)
Rearfoot EVE/INV and knee ADD/ABD	0.94* (0.57)	0.83* (0.71)	0.52*** (0.63)
Rearfoot EVE/INV and tibial INR/EXR	0.82* (0.33)	0.80* (0.36)	0.86* (0.55)
Rearfoot PRO/SUP and knee FLX/EXT	− 0.92* (0.02)	− 0.94* (0.02)	− 0.06***** (0.11)
Rearfoot PRO/SUP and knee ADD/ABD	− 0.85* (0.56)	− 0.94* (0.74)	− 0.49*** (0.59)
Rearfoot PRO/SUP and tibial INR/EXR	− 0.86* (0.20)	− 0.82* (0.27)	− 0.29**** (0.46)
Ankle DOR/PLAN and knee FLX/EXT	0.83* (0.04)	0.86* (0.01)	0.16***** (0.08)

All were significant (*P* < 0.001). Negative values: inverse correlation; positive values: positive correlation. SLL: single-leg landing; DLL: double-leg landing. Strength of correlations: *: Very strong, **: Strong, ***: Moderate, ****: Weak, *****: Very weak

### Double leg landing

Rearfoot EVE/INV was strongly correlated with hip ADD/ABD (*P* < 0.001 for all variables). Rearfoot EVE/INV and PRO/SUP had very strong correlations with the rest of the kinematic outputs of the knee and hip joints. Moreover, there was a strong correlation between ankle DOR/PLAN and hip FLX/EXT, while the rest of the correlations between rearfoot (EVE/INV and PRO/SUP) and hip FLX/EXT, and rearfoot (DOR/PLAN, EVE/INV and PRO/SUP) and knee FLX/EXT were very strong during DLL (Table 1).

### Gait

Rearfoot EVE/INV had moderate correlations with hip and knee ADD/ABD (*P* < 0.001 for all variables). Additionally, rearfoot PRO/SUP showed moderate correlations with hip ADD/ABD and tibial INR/EXR. Both rearfoot EVE/INV and INR/EXR were strongly correlated with hip INR/EXR. Rearfoot EVE/INV had a very strong correlation with tibial INR/EXR. Moreover, weak correlation between rearfoot PRO/SUP and hip FLX/EXT, and very weak correlation between ankle DOR/PLAN and knee FLX/EXT were observed, while moderate correlations were observed between rearfoot EVE/INV and hip and knee FLX/EXT, and rearfoot PRO/SUP and knee FLX/EXT. The only strong correlation during walking was observed between ankle DOR/PLAN and hip FLX/EXT (Table 1).

Further information on correlation coefficients and coupling angles is provided in Tables 1 and 2. The angular displacements over the period from initial contact to maximum landing and frequency diagrams of the correlation strengths are provided in the supplementary material.

**Table 2:** Correlation angles in degree mean value + (SD) during different tasks in sagittal, frontal and transverse motion planes

Couplings angles (°)	SLL N = 26	DLL N = 26	Gait N = 26
Rearfoot EVE/INV and hip FLX/EXT	25.52 (2.04)	13.86 (1.19)	16.9 (2.33)
Rearfoot EVE/INV and hip ADD/ABD	31.4 (10.4)	36.0 (7.8)	30.6 (11.0)
Rearfoot EVE/INV and hip INR/EXR	29.0 (11.1)	28.7 (11.4)	27.5 (10.5)
Rearfoot PRO/SUP and hip FLX/EXT	50.06 (1.44)	35.8 (1.58)	40.66 (2.09)
Rearfoot PRO/SUP and hip ADD/ABD	57.2 (6.4)	62.8 (6.54)	60.5 (6.7)
Rearfoot PRO/SUP and hip INR/EXR	54.9 (7.6)	54.4 (8.7)	57.2 (7.1)
Ankle DOR/PLAN and hip FLX/EXT	50.23 (1.7)	44.21 (1.62)	42.51 (1.52)
Rearfoot EVE/INV and knee FLX/EXT	19.37 (1.71)	8.51 (0.76	19.16 (1.49)
Rearfoot EVE/INV and knee ADD/ABD	27.4 (10.0)	24.6 (11.3)	39.4 (12.48)
Rearfoot EVE/INV and tibial INR/EXR	20.7 (6.6)	15.8 (5.8)	20.0 (6.3)
Rearfoot PRO/SUP and knee FLX/EXT	42.43 (1.44)	26.08 (1.0)	46.96 (1.4)
Rearfoot PRO/SUP and knee ADD/ABD	53.2 (7.4)	51.1 (9.1)	67.6 (6.2)
Rearfoot PRO/SUP and tibial INR/EXR	47.8 (6.0)	41.6 (7.7)	49.5 (7.2)
Ankle DOR/PLAN and knee FLX/EXT	41.5 (0.97)	33.1 (0.92)	48.61 (1.93)

These angles represent smaller or greater coupling variability. SLL: single-leg landing; DLL: double-leg landing. All values are in degrees

### Between tasks comparison

A very strong correlation during all three tasks was observed between rearfoot EVE/INV and tibial INR/EXR only (*P* < 0.001). Rearfoot EVE/INV had moderate to strong correlations with hip ADD/ABD in all the tasks (*P* < 0.001). The strongest correlations between rearfoot, knee, and hip joints were observed during the double-leg landing and single-leg landing (*P* < 0.001). In contrast, such correlations were typically not as strong during gait (*P* < 0.001). The correlation between rearfoot PRO/SUP and hip ADD/ABD was very strong during double-leg landing, weak during single-leg landing, and moderate during gait (*P* < 0.001). Rearfoot EVE/INV and knee ADD/ABD were moderately correlated during gait and strongly correlated during single and double leg landings (*P* < 0.001) (Table 1). The correlations were moderate to weak during gait, strong to very strong during SLL, and very strong (except for ankle DOR/PLAN and hip FLX/EXT which was strong) during DLL (*P* < 0.001).

## Discussion

The current study found that the strongest correlations existed during the DLL task, while less strong correlations were observed during gait. The correlation between rearfoot EVE/INV and tibial INR/EXR was the strongest in all the physical tasks in this study. Interestingly, weak to moderate coupling between rearfoot EVE/INV and INR/EXR and hip ADD/ABD, and very weak correlation between ankle DOR/PLAN and knee FLX/EXT were observed during gait.

Foot and knee rotational motions are related to each other in participants with knee valgus [13]. Additionally, comparing coupling variability between genders showed that females may generate stronger coupling between the knee and hip motions in frontal and transverse planes [23]. Our findings were consistent with the results of a study in which the strongest coupling was observed between rearfoot and knee and hip joints during single-leg landing, compared to the gait [20]. However, in that study, strong correlations were reported between rearfoot EVE/INV and hip ADD/ABD as well as between rearfoot PRO/SUP and hip ADD/ABD, whereas in our study, strong correlations were observed between rearfoot and knee motions in frontal and horizontal planes and hip joint in horizontal plane. The difference in findings could have stemmed from differences in gender and skeletal development of participants between the two studies. The previous study assessed kinematic coupling in healthy male and female adults, whereas the sample in our study consisted of female athletes during puberty with DKV. Greater knee-dominant landing strategies observed in female athletes compared to males [51], altered biomechanical characteristics such as greater hip internal rotation and adduction, knee abduction and external rotation, foot pronation, and limited ankle dorsiflexion range of motion observed in participants exhibiting DKV compared to the healthy counterparts [52, 53], and lower rates of neuromuscular control in the lower extremity inducing greater knee abduction angle and moment in female athletes during puberty may be the underlying reasons behind the observed differences in the strength of couplings [34].

The outcomes of the current study regarding the positive correlations between greater rearfoot EVE and knee ABD are in contrast to another study, which reported decreased rearfoot EVE in response to excessive knee ABD [16]. Greater foot pronation affecting rearfoot eversion observed in younger participants may explain this difference [54]. Unlike the previous study reporting a trivial relationship between rearfoot and knee joint’s kinematics, and strong relationships between rearfoot and hip motions [55], our results imply a strong coupling between rearfoot and knee during single and double-leg landings. The evidence on the biomechanical link between rearfoot EVE/INV and tibial rotations remains contradictory. A study claimed that foot EVE is the major contributor to tibial and femoral INR during gait [55]. In contrast, another study did not find strong links between rearfoot EVE/INV and tibial rotation in the gait cycle [49].

Additionally, very strong correlations existed between hip and knee FLX/EXT and ankle PRO/SUP and EVE/INV, except for hip FLX/EXT and ankle DOR/PLAN during DLL where the correlation was strong. During SLL, the correlations were very strong between all the coupled motions, while ankle correlations with hip and knee joints at the sagittal plane were strong. This can indicate higher reliance on hip and knee joints to compensate for alternations in rearfoot motion in frontal and transverse planes [56]. However, weak to moderate correlations between hip and knee joints at the sagittal plane and ankle/rearfoot motions at sagittal, frontal and transverse planes during gait compared to strong to very strong results in SLL and DLL tasks suggests that kinematic coupling is task-dependent. A previous study reported that women had less coupling variability between knee FLX/EXT and hip and knee rotational motions during an unanticipated cutting maneuver compared to their male counterparts [23]. However, to the best of our knowledge, no study has investigated the sagittal plane coupling kinematics with frontal and transverse planes between lower extremity joints during landing and gait in female athletes with DKV or during pubertal growth. Based on our findings, greater kinematic coupling can be observed between knee and ankle joints during SLL and DLL, while the greatest coupling variability is expected during DLL.

Compensatory motions in female athletes with DKV during puberty were found between hip and knee motions in sagittal plane and ankle joint frontal and transverse planes during landing tasks. It has been suggested that greater reliance on hip and knee joints at frontal and transverse planes in response to limited ankle motions at sagittal plane allows athletes to lower the body center of mass in order for a better ground reaction force attenuation [57]. The motions of each joint in relation to its proximal and distal joints observed at sagittal, frontal, and transverse planes may be the underlying reason behind the biomechanical link between limited ankle dorsiflexion range of motion in participants with knee valgus putting them at higher risk of ACL injuries [58, 59]. However, based on our results, gait is not recommended as a proper task for evaluation of the full potential of lower extremity joint couplings due to considerably weaker correlations observed between all three motion planes compared to the correlations during more physically demanding tasks.

Based on the outcomes of the current study, coupling patterns did not show to be consistent during single and double-leg landing and gait tasks. This task-related difference may be due to the different intensity levels between these tasks, changing biomechanical behavior. For instance, greater knee abduction motion is observed during more physically demanding tasks such as jumping-landings than walking [19]. Thus, we suggest clinicians consider task intensity levels and the possible presence of biomechanical abnormalities in assessing couplings during different activities.

Generally, it is expected to observe a greater range of motion in the foot during intense physical activities, and consequently, increased kinematic coupling between the involved joints [19]. Previous studies found rearfoot pronation to influence function and injury susceptibility in proximal joints [14]. Further research is needed on this matter in participants with imbalances such as DKV. Additionally, coaches and clinicians should concentrate on athletes’ kinematic coupling variability since it plays a prominent role in ACL injury occurrence [30, 31], increases susceptibility to patellofemoral pain syndrome [60], and may even be associated with mechanical lower back pain [61, 62].

So far, a few mechanisms have been suggested to be responsible for the differences in coupling strength between rearfoot and knee and hip joints during different tasks. It is believed that greater hip INR may be generated as a protective and compensatory movement to keep the knee joint at a more stable and less rotated condition during intense physical activities such as jump-landings [60]. Additionally, joint motions are concomitantly affected by their surrounding muscles. Biomechanical studies showed that muscular function could affect the biomechanical relationships in multi-joint systems like lower extremities [63]. Therefore, it can be postulated that as the muscles’ activity increases in more physically demanding tasks. Regarding probable strong coupling patterns in participants with DKV, weaker muscular control comes in mind, as lower strength in gluteus maximus and hip abductors are associated with greater knee valgus alignment [64, 65]. Thus, employing plyometric and neuromuscular exercises that enhance muscular activation would benefit the athletes by possibly minimizing the abnormal coupling patterns, for instance adopting a knee-bias strategy, which collectively contribute to reducing the risk of injuries [27, 66]. Based on the results of the current study, we suggest clinicians and coaches to consider relative motions between two joints in the same or different motion planes when evaluating injurious biomechanical characteristics. Moreover, the exercise protocols aimed at mitigating the risk of ACL injury should be designed to address the lower extremity joints as a whole chain, instead of focusing on the motions of one joint only.

One of the study limitations is the inclusion of female athletes between the ages of 10 to 14 only, which limits generalizing the results of this study to male athletes or other age brackets. Further studies on male athletes are needed, as lower extremity motion patterns vary between female and male athletes [67–69]. Moreover, the results of the current study cannot be generalized to older female athletes. Regarding the tasks, participants of the current study performed gait task at their preferred speed and landed from a box set at fixed height. However, future studies need to evaluate the probable effect of walking speed on kinematic coupling. Moreover, since jump-landing task is different in the sports fields, kinematic cooling should be evaluated during landing tasks from a higher surface. As another limitation, we did not consider the effect of muscle force on kinematic coupling which is required to be further analyzed. Maybe the fact that only gait, SLL and DLL were evaluated in a controlled environment. Un-anticipated and more physically demanding sport-specific tasks may reveal new information about the couplings.

## Conclusions

In conclusion, the strongest kinematic coupling between rearfoot and knee and hip joints was observed during double leg landing, and with less strength between rearfoot EVE/INV and PRO/SUP and hip ADD/ABD during single leg landing task. In another words, this indicated greater movement variability during landing tasks. Additionally, rearfoot EVE/INV and tibial INR/EXR had the most rigidly linked relationship during single-leg landing, double-leg landing, and gait. Thus, the rotational motions of tibia were affected by rearfoot EVE/INV, in tasks with different intensity and biomechanics. However, due to the differences in kinematic coupling strength between the tasks, the current study results suggest that coupling relationships vary by task intensity in female junior athletes with DKV.

## Data Availability

The raw data and material will be shared after a maximum of one week of request to the corresponding author.

## Abbreviations

DKV: Dynamic Knee Valgus
ACL: Anterior Cruciate Ligament
CM: Centimeter
SLL: Single-leg-landing
DLL: Double-leg-landing
IC: Initial Contact
MAX: Maximum Landing
VGRF: Vertical Ground Reaction Force
EVE/INV: Eversion/Inversion
FLX/EXT: Flexion/Extension
ADD/ABD: Adduction/Abduction
PRO/SUP: Pronation/Supination
DOR/PLAN: Dorsiflexion/Plantarflexion
MATLAB: Matrix Laboratory
SD: Standard deviation
